# Clinical care in hepatocellular carcinoma: A mixed methods assessment of experiences and challenges of oncology professionals

**DOI:** 10.1002/cam4.5216

**Published:** 2022-09-15

**Authors:** Ginny Jacobs, Deborah A. Boyle, Hashem B. El‐Serag, Robert J. Lewandowski, Stacey M. Stein, Patrice Lazure, Pam McFadden

**Affiliations:** ^1^ AXDEV Global Inc. Virginia Beach Virginia USA; ^2^ Advanced Oncology Nursing Resources Phoenix Arizona USA; ^3^ Texas Medical Center Digestive Diseases Center Houston Houston Texas USA; ^4^ Northwestern Memorial Hospital, Department of radiology Chicago Illinois USA; ^5^ Yale School of Medicine New Haven Connecticut USA; ^6^ AXDEV Group Inc. Brossard Quebec Canada

**Keywords:** cancer management, hepatocellular carcinoma, liver cancer, medical oncology, screening

## Abstract

**Introduction:**

Healthcare providers (HCPs) may face numerous dilemmas in optimally screening, diagnosing, and treating patients with, and/or at risk for, hepatocellular carcinoma (HCC). This study aimed to achieve a greater understanding of the challenges in HCC care which in turn could delineate HCP educational opportunities within this oncologic sub‐specialty.

**Methods:**

A mixed‐methods approach was used to identify practice gaps and clinical barriers experienced by US‐based medical oncologists, hepatologists, oncology physician assistants, oncology nurse practitioners, and interventional radiologists involved in HCC care. The qualitative (semi‐structured interview) and quantitative (survey) data collection approaches were deployed sequentially with findings subsequently triangulated.

**Results:**

A total of 214 HCPs participated in this study. Analysis revealed challenges related to screening and diagnosing HCC, specifically in applying appropriate screening guidelines, and the optimal use and decisions related to diagnostic imaging and biopsy. Issues related to treatment selection included the application of existing HCC guidelines in treatment decision‐making, weighing risk/benefit ratios of various antineoplastics regimens (i.e., tyrosine kinase inhibitors‐TKIs, immunotherapy agents, chemotherapy), sequencing therapies, potential toxicity management, and optimally educating patients about their HCC.

**Conclusion:**

These findings highlight the educational needs of those involved in HCC care and provide a starting point for clinicians to both reflect on their practice and identify opportunities to enhance communication within the HCC team and between provider and patient. There is an opportunity to optimize continuing professional development interventions that address the identified gaps in clinical practice specifically related to teamwork and interdisciplinary communication.

## Graphical abstract

This mixed‐methods study assessed the challenges faced by healthcare professionals (HCP) involved in the care ofpatients with hepatocellular carcinoma (HCC). Interviews and surveys with HCPs from across the United States indicatedchallenges applying screening and treatment guidelines, diagnosing HCC via imaging and biopsy, selecting andsequencing treatments, managing toxicities and educating patients about HCC. The findings from this needs assessmentwill help identify gaps that exist in knowledge, competence, and skill, so that subsequent educational interventions canbe designed to promote effective collaborative care and encourage patient engagement.

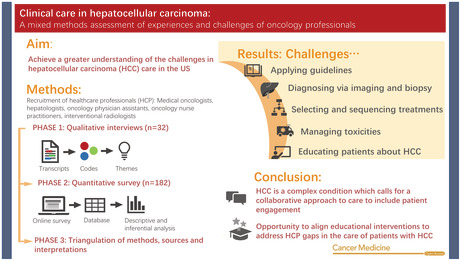

## 1 INTRODUCTION

Primary liver cancer is a leading cause of cancer death worldwide.^1^ Hepatocellular carcinoma (HCC) represents 75%–85% of primary hepatic malignancies.^2^ Its occurrence has increased globally over the last decades and is projected to affect 1.4 million people by 2040.^3^, ^4^ Risk factors such as hepatitis B and C, chronic alcohol consumption, and non‐alcoholic fatty liver disease (NAFLD) are associated with HCC resulting from cirrhosis.^5^, ^6^ Currently, NAFLD's limited diagnostic testing options and therapeutic modalities increase HCC's global prevalence.^7^

While early diagnosis is associated with improved outcomes and disease control, HCC tends to be diagnosed at a later stage because of low disease awareness from general practitioners and patients, low levels of screening of at‐risk patients by specialists, and the high associated cost of screening.^8^, ^9^ In particular, appropriate screening (i.e., with ultrasound, non‐invasive cross‐sectional abdominal imaging or alpha fetoprotein [AFP] marker testing) is underutilized for patients at risk of HCC. This often results in a diagnosis of advanced stage HCC when liver resection, transplantation, or ablation are no longer viable treatment options.^10^, ^11^ Biopsy‐guided diagnosis also has limitations due to access (e.g., obscured tumor location, presence of ascites) and concern for increased risk of bleeding.^12^, ^13^

The use of systemic therapies for patients with advanced HCC, especially those not candidates for resection, transplantation, or local ablative therapies, have recently increased.^14^, ^15^ However, toxicity prevalence has been associated with dose reduction or treatment interruption.^10^ Due to the plethora of novel systemic therapy options for HCC, new challenges for HCPs have arisen related to staying updated with those rapidly evolving treatment options, and the ideal regimen sequencing.^16^

The complexity and heterogeneity of HCC requires treatment selection to be highly individualized and, ideally, multidisciplinary in nature, integrating numerous indices such as tumor burden and stage, performance status, presence of comorbidities, quality of life (QoL), and patient preferences.^17^, ^18^ However, such multidisciplinary settings are not uniformly available, and while palliative care is ideally integrated into care planning early in the patient trajectory, unfortunately, these services are currently underutilized for patients with HCC.^19^

As a cancer with increasing global incidence that has a propensity for late‐stage diagnosis and currently lacks effective curative treatment options, there is a need to better understand and address potential challenges that HCC healthcare providers (HCPs) face. HCPs who are well‐informed and who more effectively leverage the knowledge and experience of their colleagues (and coordinate their care delivery as a member of the HCP team) are better able to inform the patient regarding their care pathway.

The primary objective of this study was to identify and categorize team‐based clinical practice gaps and challenges experienced by US‐based medical oncologists (MOs), hepatologists (HEPs), oncology physician assistants (PAs), oncology nurse practitioners (NPs), and interventional radiologists (IRs) involved in the care of patients with HCC. The identification of these issues can provide a starting point for clinicians to reflect on their practice and for both clinicians and educators to recognize opportunities for learning and improvement.

## 2 METHODS

A mixed‐methods sequential design combining a qualitative exploratory data collection phase with a quantitative validation phase was utilized.^20^ The qualitative exploration consisted of 45‐minute semi‐structured interviews with open‐ended questions to explore the HCP's perceptions about screening, diagnosis, and treatment of HCC. Additionally, participants were queried about the nature and quality of their inter‐provider and patient‐provider communication. Findings from this qualitative phase informed the development of a 20‐min online survey (quantitative validation phase). The study was approved by Veritas IRB (QC, Canada), an international, independent ethical review board registered with the United States Department of Health and Human Services (DHHS).

### 2.1 Recruitment

Potential participants were initially identified using two separate ICC/ESOMAR guideline compliant panels.^21^ Each panel contains validated healthcare professionals who volunteered to receive study invitations via email. A subsample of each panel, restricted to the targeted professions and specialty, was sent an email invitation containing a secure link to an online screener.^21^ Responses provided to the online screener were used to determine eligibility (see next paragraph), and only eligible participants were presented the consent form. Those who consented to participate were redirected either to the interview availability form or the online survey. Participants were compensated in accordance with the nature of their participation (interview/survey) and their profession in alignment with best practices and ethics.^22^

### 2.2 Research criteria

Eligibility criteria was established in accordance with the project focus and objectives through discussions between education experts (including GJ, PL, PM) and clinical oncology practitioners (DAB, HBE, RJL, SMS). Participants were required to have an active practice for a minimum of 3 years as a medical oncologist (MO), hepatologist (HEP), oncology PA or NP, or interventional radiologist (IR) in the United States, with a minimum monthly caseload of 10 HCC patients for MO/HEP, 3 for PA/NP, and 5 for IR. A purposive sampling methodology was used to ensure that participants represented multiple perspectives in terms of practice settings (i.e., academic, community), years of practice, and geographic location, (i.e., rural, suburban, urban).^23^

### 2.3 Data collection

A non‐exhaustive review of the literature, in tandem with consultations between education experts and clinical oncology practitioners, guided the development of the qualitative data collection instrument. The semi‐structured interview guide included open‐ended questions and probes for trained interviewers to elicit elaboration on reported professional challenges. The interviews (conducted March to May 2020) were recorded with participant consent and then transcribed. An online survey, informed by the qualitative findings, was subsequently developed and administered (August–September 2020). The survey contained 146 to 149 items (depending on profession) and was designed to evaluate participants' level of knowledge, skills, confidence, and agreement to statements specific to HCC care. A five‐point Likert‐type scale was used to quantify knowledge and skill levels (1 = no knowledge/skill; 5 = expert knowledge/skill). Confidence items utilized a visual analogue scale (0 = not at all confident; 100 = highly confident). Agreement items included a five‐point Likert‐type scale (1 = strongly disagree; 5 = strongly agree). Each item also included the option to respond, “Not relevant in my current role.”

### 2.4 Analysis

Interview transcripts were coded and analyzed through an approach drawing from the tenets of directed content analysis^24^ and thematic analysis^25^ with NVivo software (QSR International Pty Ltd, Version 12, 2018). The coding tree was developed a priori based on the interview guide structure and then refined as details emerged from the data. Quantitative data from the survey were analyzed through cross‐tabulations, analysis of variance (ANOVA), and Kruskal‐Wallis H tests with SPSS 26.0 software (IBM Corporation, Armonk, NY) to identify differences in knowledge, skills, confidence, and agreement levels according to profession, years of practice, and type of setting. The five‐point Likert‐type scale for knowledge/skill items was recoded into 2 categories: “sub‐optimal” (1 = none, 2 = basic, 3 = intermediate) and “optimal” (4 = advanced, 5 = expert).” When over 30% of a respondent sub‐group reported “sub optimal” knowledge or skills, this was considered an indication of a gap or educational need. Confidence levels were determined to be “sub optimal when the mean was below 80. The five‐point Likert scale for agreement items was recoded in 3 categories: “disagree/strongly disagree,” “neither agree nor disagree,” and “agree/strongly agree.” Qualitative and quantitative data were then combined using a triangulation of sources (five professions), methods (qualitative, quantitative), and investigational perspectives (multidisciplinary interpretation between educational and clinical experts).^20^

## 3 RESULTS

A total of 214 HCPs participated in this study: MOs (*n*_qual_ = 8; *n*_quant_ = 48), HEPs (*n*_qual_ = 8; *n*_quant_ = 45), oncology PAs (*n*_qual_ = 2; *n*_quant_ = 19), oncology NPs (*n*_qual_ = 6; *n*_quant_ = 26), and IRs (*n*_qual_ = 8; *n*_quant_ = 44). The majority (62%) of survey respondents practiced in community settings, while qualitative phase participants were evenly split between academic and community settings. Participants were generally well distributed across the US, although the West region was slightly underrepresented. Demographic data of participants are detailed in Table 1. Mixed method analysis revealed three challenges reported by the HCC team related to screening and diagnosis and five challenges related to treatment selection and management.

**TABLE 1:** Description of sample by phase (qualitative and quantitative) and speciality

Qualitative interviews	ONC (*n* = 8)	HEP (*n* = 8)	PA (*n* = 2)	NP (*n* = 6)	IR (*n* = 8)	Total (*n* = 32)
Years of practice						
3–10 years	3	1	0	5	4	13
11–20 years	4	4	2	1	4	15
21+ years	1	3	0	0	0	4
Setting						
Academic	4	3	0	4	5	16
Community	4	5	2	2	3	16
Location						
Rural	2	0	0	4	0	6
Suburban	5	4	1	2	3	15
Urban	1	4	1	0	5	11
US Region^a^						
Northeast	1	5	1	2	2	11
Midwest	3	2	0	3	3	11
South	3	0	1	1	2	7
West	1	1	0	0	1	3

Quantitative survey	ONC (*n* = 48)	HEP (*n* = 45)	PA (*n* = 19)	NP (*n* = 26)	IR (*n* = 44)	Total (*n* = 182)
Years of practice						
3–10 years	15	13	10	16	19	73
11–20 years	24	23	6	8	18	79
21+ years	9	9	3	2	7	30
Setting						
Academic	16	13	9	17	15	70
Community	32	32	10	9	29	112
Location						
Rural	3	4	2	2	1	12
Suburban	18	17	5	6	20	66
Urban	27	24	12	18	23	104
US Region^a^						
Northeast	15	12	9	11	15	62
Midwest	12	8	4	8	10	42
South	15	20	5	4	13	57
West	6	5	1	3	6	21

^a^ US Regions based on the US Census bureau (https://www2.census.gov/geo/pdfs/maps‐data/maps/reference/us_regdiv.pdf). Northeast = Connecticut, Maine, Massachusetts, New Hampshire, New Jersey, New York, Pennsylvania, Rhode Island, and Vermont. Midwest = Illinois, Indiana, Iowa, Kansas, Michigan, Minnesota, Missouri, Nebraska, North Dakota, Ohio, South Dakota and Wisconsin. South = Alabama, Arkansas, Delaware, District of Columbia, Florida, Georgia, Kentucky, Louisiana, Maryland, Mississippi, North Carolina, Oklahoma, South Carolina, Tennessee, Texas, Virginia, and West Virginia. West = Alaska, Arizona, California, Colorado, Hawaii, Idaho, Montana, Nevada, New Mexico, Oregon, Utah, Washington and Wyoming.

### 3.1 Screening and diagnosis

#### 3.1.1 Applying appropriate screening criteria/guidelines

Quantitative data revealed that 40% of all HCPs “agreed/strongly agreed” with the statement, “*There is a lack of reliable guidelines on HCC screening*,” with variability in agreement according to profession (Table 4‐A). Suboptimal knowledge of the AASLD screening guidelines for HCC was reported by most ‐ PAs (63%), NPs (69%), and IRs (65%) (Table 2‐A). Regarding the NCCN screening guidelines for HCC, suboptimal knowledge was also reported by most—HEPs (58%), PAs (58%), NPs (50%), and IRs (65%) (Table 2‐B). Similarly, suboptimal knowledge was also reported regarding the NCI PDQ HCC screening guidelines by the majority of HEPs (60%), PAs (74%), NPs (69%), and IRs (70%) (Table 2‐C).

**TABLE 2:** Percent of HCPs who self‐reported no or basic levels of knowledge or skills

Percent of ……… who reported no or basic…		Profession					Total	Sig.^a^
		ONC	HEP	PA	NP	IR		
A	..... knowledge of AASLD screening guidelines (Diagnosis, Staging, and Management of HCC: 2018 Practice Guidance by the AASLD)	44% (*n* = 21)	22% (*n* = 10)	63% (*n* = 12)	69% (*n* = 18)	65% (*n* = 28)	49% (*n* = 89)	*p* < 0.001
B	..... knowledge of NCCN screening guidelines (NCCN Guidelines® for Hepatobiliary Cancers)	33% (*n* = 16)	58% (*n* = 26)	58% (*n* = 11)	50% (*n* = 13)	65% (*n* = 28)	52% (*n* = 94)	*p* < 0.05
C	..... knowledge of NCI PDQ screening guidelines (Liver (Hepatocellular) Cancer Screening (PDQ®))	44% (*n* = 21)	60% (*n* = 27)	74% (*n* = 14)	69% (*n* = 18)	70% (*n* = 30)	61% (*n* = 110)	*p* = 0.052
D	..... knowledge of contraindications for imaging modalities	34% (*n* = 16)	29% (*n* = 13)	58% (*n* = 11)	46% (*n* = 12)	5% (*n* = 2)	30% (*n* = 54)	*p* < 0.001
E	..... knowledge of potential side effects of tyrosine kinase inhibitors	27% (*n* = 13)	53% (*n* = 24)	58% (*n* = 11)	54% (*n* = 14)	88% (*n* = 36)	55% (*n* = 98)	*p* < 0.001
F	..... knowledge of potential side effects of immunotherapy agents	25% (*n* = 12)	58% (*n* = 26)	32% (*n* = 6)	31% (*n* = 8)	85% (*n* = 35)	49% (*n* = 87)	*p* < 0.001
G	..... knowledge of potential side effects of conventional chemotherapy agents	27% (*n* = 13)	44% (*n* = 20)	21% (*n* = 4)	27% (*n* = 7)	81% (*n* = 34)	43% (*n* = 78)	*p* < 0.001
H	..... knowledge of best practices to treat HCC in patients with each of the following comorbid renal failure	42% (*n* = 20)	60% (*n* = 27)	63% (*n* = 12)	69% (*n* = 18)	70% (*n* = 30)	59% (*n* = 107)	*p* < 0.05
I	..... knowledge of best practices to treat HCC in patients with each of the following comorbid lung disease	31% (*n* = 15)	60% (*n* = 27)	74% (*n* = 14)	65% (*n* = 17)	81% (*n* = 34)	59% (*n* = 107)	*p* < 0.001
J	..... knowledge of best practices to treat HCC in patients with each of the following comorbid cardiovascular disease	33% (*n* = 16)	62% (*n* = 28)	63% (*n* = 12)	62% (*n* = 16)	72% (*n* = 31)	57% (*n* = 103)	*p* < 0.05
K	..... knowledge of best practices in treatment sequencing	29% (*n* = 14)	56% (*n* = 25)	68% (*n* = 13)	58% (*n* = 15)	74% (*n* = 31)	54% (*n* = 98)	*p* < 0.001

Abbreviations: HEP, Hepatologists; IR, Interventional radiologists; NP, Nurse practitioners; ONC, Medical oncologists; PA, Physician assistants.

a Chi‐squared test.

Across all professional groups, an average of 78% of all HCPs “agreed/strongly agreed” that, “*There is a lack of awareness about HCC screening at the primary care level”* (Table 4‐B), a concern that HCPs also expressed during the qualitative phase (Table 6‐Q1).

PAs (47%), NPs (46%), and IRs (51%) reported sub‐optimal skills determining the need for ongoing surveillance following a first screening for HCC (Table 3‐A). HCPs also reported suboptimal mean levels of confidence in developing an action plan based upon the results of screening: between (mea*n* ± standard deviation; 0–100 scale) 64 ± 20 for PAs and 78 ± 19 for HEPs (Table 5‐A).

**TABLE 3:** Percent of HCPs who self‐reported no or basic levels skills

Percent of … … who reported no or basic…		Profession					Total	Sig.*
		ONC	HEP	PA	NP	IR		
A	*…* skill in determining the need for ongoing surveillance following first screening	25% (*n* = 12)	18% (*n* = 8)	47% (*n* = 9)	46% (*n* = 12)	51% (*n* = 22)	35% (*n* = 63)	*p* < 0.05
B	… skill in interpreting imaging results with atypical presentation	43% (*n* = 20)	38% (*n* = 17)	72% (*n* = 15)	92% (*n* = 24)	23% (*n* = 10)	48% (*n* = 86)	*p* < 0.001
C	… skill in determining which cases necessitate a biopsy of suspicious lesion/mass	27% (*n* = 13)	16% (*n* = 7)	47% (*n* = 9)	54% (*n* = 14)	14% (*n* = 6)	27% (*n* = 49)	*p* < 0.001
D	… skill in identifying the safest and most effective treatment option for a specific patient	17% (*n* = 8)	38% (*n* = 17)	53% (*n* = 10)	54% (*n* = 14)	62% (*n* = 26)	42% (*n* = 75)	*p* < 0.001
E	… skill in managing toxicities to enhance quality of life (QoL)	17% (*n* = 8)	53% (*n* = 24)	37% (*n* = 7)	23% (*n* = 6)	82% (*n* = 32)	44% (*n* = 77)	*p* < 0.001
F	… skill in making treatment decisions for patients with multiple comorbidities	21% (*n* = 10)	47% (*n* = 21)	58% (*n* = 11)	46% (*n* = 12)	70% (*n* = 28)	46% (*n* = 82)	*p* < 0.001
G	… skill in determining the optimal sequencing of treatments	27% (*n* = 13)	38% (*n* = 17)	74% (*n* = 14)	54% (*n* = 14)	73% (*n* = 30)	49% (*n* = 88)	*p* < 0.001
H	… skill in promoting realistic expectations about chosen treatment option	29% (*n* = 14)	29% (*n* = 13)	42% (*n* = 8)	23% (*n* = 6)	57% (*n* = 25)	36% (*n* = 66)	*p* < 0.05

Abbreviations: HEP, Hepatologists; IR, Interventional radiologists; NP, Nurse practitioners; ONC, Medical oncologists; PA, Physician assistants.

^*^ Chi‐squared test.

#### 3.1.2 Using imaging for diagnosis

MOs (34%), PAs (58%), and NPs (46%) reported sub‐optimal knowledge of contraindications for imaging modalities (Table 2‐D). Qualitative data also revealed how patient‐specific variables can make the diagnosis challenging (Table 6‐Q2).

Suboptimal skills interpreting imaging results with atypical presentations were reported by MOs (43%), HEPs (38%), PAs (79%), and NPs (92%) (Table 3‐B). Interviewees shared that initial imaging results do not always meet the HCC criteria such as in the case of patients having atypical features, which renders the diagnosis difficult to establish (Table 6‐Q3).

#### 3.1.3 Biopsy decision‐making

Twenty‐seven percent of MOs, 47% of PAs, and 54% of NPs reported suboptimal skills determining which cases necessitate a biopsy of a suspicious lesion/mass (Table 3‐C). Suboptimal confidence when deciding if a biopsy of a suspicious lesion/mass is required was also reported by MOs (74 ± 16), PAs (66 ± 19), and NPs (62 ± 22) (Table 5‐B). Qualitative data characterized the difficulties experienced when imaging findings were inconclusive (Table 6‐Q4).

### 3.2 Treatment selection and management

#### 3.2.1 Applying guidelines in treatment decisions

On average, 44% of HCPs “agreed/strongly agreed” that, “*Treatment decisions for HCC are difficult because there are too many options*” (Table 4‐C). Interviewed participants reported that existing guidelines do not always optimally support decision‐makers in considering multiple treatment options (Table 6‐Q5/Q6).

**TABLE 4:** Percent of HCPs who agreed or strongly agreed with key opinion statements

Percent of ...... who agreed or strongly agreed with the statement:		Profession					Total	Sig.*
		ONC	HEP	PA	NP	IR		
A	“There is a lack of reliable guidelines on HCC screening”	52% (*n* = 25)	29% (*n* = 13)	58% (*n* = 11)	42% (*n* = 11)	28% (*n* = 12)	40% (72)	*p* < 0.05
B	“There is a lack of awareness about HCC screening at the primary care level”	75% (*n* = 36)	80% (*n* = 36)	68% (*n* = 13)	80% (*n* = 20)	81% (*n* = 35)	78% (*n* = 140)	*p* = 0.84
C	“Treatment decisions for HCC are difficult because there are too many options”	50% (*n* = 24)	60% (*n* = 27)	32% (*n* = 6)	35% (*n* = 9)	33% (*n* = 14)	44% (*n* = 80)	*p* = 0.196

Abbreviations: HEP, Hepatologists; IR, Interventional radiologists; NP, Nurse practitioners; ONC, Medical oncologists; PA, Physician assistants.

^*^ Chi‐squared test.

#### 3.2.2 Balancing risks and benefits of treatment

Quantitative survey data revealed suboptimal knowledge of potential side effects of TKIs (55%), immunotherapeutics (49%), and conventional chemotherapy agents (43%), with variation between profession groups (Table 2‐E/F/G). Interviewees also expressed difficulty finding relevant information on side effect prevalence for newer therapy options (Table 6‐Q7).

HEPs (38%), PAs (53%), NPs (54%), and IRs (62%) reported suboptimal skills identifying the safest and most effective treatment option for a specific patient (Table 3‐D). Interviewees expressed the perception that the toxicities of newer agents seem to generally outweigh the benefits of their efficacy (Table 6‐Q8).

Suboptimal skills managing toxicities to enhance QoL were also reported by HEPs (53%), PAs (37%), and IRs (82%) (Table 3‐E). Confidence in making the necessary treatment changes to reduce side effects was suboptimal for all professions, ranging from 42 ± 31 for IRs to 75 ± 18 for MOs (Table 5‐C). Qualitative data reveal concerns about using systemic therapy because of the side effects that can be difficult to tolerate and may lead to non‐adherence (Table 6‐Q9).

**TABLE 5:** Self‐reported confidence levels to selected items

Mean confidence level and std. deviation when…		Profession					Total	Sig.^a^
		ONC	HEP	PA	NP	IR		
A	Developing action plan based upon results of screening	75 ± 20 (48)	78 ± 19 (45)	64 ± 20 (19)	77 ± 19 (25)	66 ± 26 (43)	73 ± 22 (180)	*p* < 0.05
B	Deciding if a biopsy of suspicious lesion/mass is required	74 ± 16 (48)	78 ± 19 (45)	66 ± 19 (19)	62 ± 22 (26)	81 ± 14 (44)	74 ± 19 (182)	*p* < 0.001
C	Making the necessary treatment changes to reduce side effects	75 ± 18 (48)	71 ± 21 (44)	67 ± 21 (19)	71 ± 23 (26)	42 ± 31 (39)	65 ± 26 (176)	*p* < 0.001
D	Communicating to patients the importance of reporting side effects	79 ± 14 (48)	79 ± 17 (44)	72 ± 20 (19)	83 ± 20 (26)	64 ± 29 (43)	75 ± 22 (180)	*p* < 0.05

Abbreviations: HEP, Hepatologists; IR, Interventional radiologists; NP, Nurse practitioners; ONC, Medical oncologists; PA, Physician assistants.

^a^ Kruskal‐Wallis H test.

**TABLE 6:** Representative quotes from the qualitative interviews

Theme	Illustrative quotes
A1. Applying appropriate screening criteria and guidelines	Q1: *“I think it's pretty laid out well for us by the AASLD about who we should be screening. I think that there is a lot of pushback. I have personally dealt with some pushback from some primary care physicians in the community that feel that it's overkill doing an ultrasound every six months for these patients. They feel it's a waste of money.”* – Hepatologist
A2. Using imaging for diagnosis	Q2: *“Some people cannot get an MRI for whatever reason*, *if it's a matter of claustrophobia or implants and things like that. And as I said*, *the concern for CT is especially if they get follow‐up on a recurring basis*, *that there's radiation exposure […] if they have contraindications for the contrast*, *that can be an issue. That would be a limitation for evaluating these cases. In which case we can still do ‐ we can get some idea about the tumors with MRI without contrast. Obviously*, *it's not ideal.”* – Interventional radiologist Q3: *“The imaging criteria are helpful*, *but they are not perfect. So*, *we are often in a situation where there is a suspicious type of legion*, *but it does not quite fulfill all the criteria to make it HCC. So that happens*, *particularly when the alpha‐fetoprotein is not elevated. That's kind of a diagnostic challenge.”* – Hepatologist
A3. Biopsy decision‐making	Q4: *“There are some patients with very atypical presentations of HCC on imaging. We do have multidisciplinary tumor boards particularly to evaluate these patients and sometimes that can be a challenge. And then*, *there's always the question of whether they need a liver biopsy or not. Particularly given the theoretical spread of the liver cells if you are getting a biopsy. I think there are some diagnostic dilemmas.”* – Hepatologist
B1. Applying guidelines in treatment decisions	Q5: *“... the explosion in systemic therapy options that have evolved in the last year or two that have really changed the landscape of systemic options. Synthesis of that information has been a little challenging as we do not quite have great guidelines on how to use some of those medications …”* – Interventional radiologist Q6: *“I try to follow the NCCN guidelines when I can. And sometimes they are helpful*, *sometimes they are less so. […] Some of it is based on the data that's available. We just do not know what's better*, *if it's better to do something local regional or if it's better to do something systemic.”* – Medical oncologist
B2. Balancing risks and benefits of treatment	Q7: *“I would say a second barrier is learning all the new side effects and management and dose suggestions for the new agents. […] I think the drug companies could have more physician‐friendly supportive materials. They're so weighed down by these legal requirements from the FDA*, etcetera, *that these websites are so tortured to navigate. They become limited in their utility. I think if the websites were a little bit more user friendly and a little bit really more helpful for the clinician*, *I would appreciate that.”* – Medical oncologist Q8: *“I think there's a lot of excitement about all of the new drugs and approvals and everything*, *but I think my challenge honestly is I do see there are a lot of new drugs available but the efficacy of all of them does not seem to warrant the degree of excitement that's out there. I think they all add something*, *and it's nice to have additional options in line for therapy*, *but I think the toxicities can be pretty significant and what we are getting out of these agents is certainly not what we would hope for.”* – Medical oncologist Q9: *“…most of these drugs that are out—whether they are the tyrosine kinase inhibitors*, *the multikinase inhibitors*, *the PD1 inhibitors—all have fairly significant side effect profiles. […] even once you have made the decision to initiate one of these patients on one drug or a combination of drugs*, *they all respond differently to the medications. Some of them have severe side effects that limit their ability to continue on that medication.”* – Interventional radiologist
B3. Patient profiles and comorbidities	Q10: *“… Surgeons are certainly more hesitant to operate on a patient who has a lot of comorbidities. Interventional radiology is hesitant to perform embolization*, *and things like that*, *on patients who are frail and do not have a good performance status. […] If they have a history of esophageal varices*, *or if they have the history of bleeding from those varices*, *then it's not going to be safe to give them*, *say*, *bevacizumab and atezolizumab because of the risk of bleeding. So*, *you really have to take it all into account. Often times*, *things will be eliminated just based on their comorbidities and the degree of their liver dysfunction*, *which limits what we can do.”* – Nurse practitioner
B4. Sequencing treatments	Q11: *“The biggest issue is trying to navigate between the TKIs and immunotherapy and the local regional therapy. To try to figure out when to do local regional*, *when to do immunotherapy*, *when to do TKIs. [...] it's sometimes tough to figure out exactly what to do when*, *in what sequence*, *in terms of what's best for the patient.”* – Medical oncologist
B5. Promoting realistic treatment expectations	Q12: *“Sometimes they do not want to talk about it. But sometimes they want to know about the details. How many months am I going to live*, *what are the chances that this works. The patients really vary. Some are very insistent on getting numbers and all the data*, *and some of them really just*, *just they do not want to talk about it. They're very anxious to talk about it.”* – Medical oncologist

#### 3.2.3 Patient profiles and co‐morbidities

HCPs reported suboptimal knowledge of best practices to treat HCC in patients with comorbid renal failure (59%), lung disease (59%), and cardiovascular disease (57%), with variations between profession groups (Table 2‐ H/I/J). Suboptimal skills making treatment decisions for patients with multiple comorbidities were also reported by HEPs (47%), PAs (58%), NPs (46%), and IRs (70%) (Table 3‐F). Interviewees expressed concerns about the heightened risks for patients with comorbidities, which limits the number of options available (Table 6‐Q10).

#### 3.2.4 Sequencing treatments

Knowledge of best practices in treatment sequencing, and skills determining the ideal sequencing of treatments were reported as suboptimal for all professions, ranging from 29% of MOs to 74% of IRs for knowledge (Table 2‐K), and from 27% of MOs to 74% of PAs for skills (Tables 3‐G and 6‐Q11).

#### 3.2.5 Promoting realistic treatment expectations

MOs (29%), HEPs (29%), PAs (42%), and IRs (57%) reported suboptimal skills promoting realistic expectations to patients about chosen treatment option (Table 3‐H). Qualitative data revealed a related perception that some patients are reluctant to discuss their long‐term treatment expectations using realistic projections (Table 6‐Q12). Suboptimal confidence was reported by PAs (72 ± 20) and IRs (64 ± 29) in communicating to patients the importance of reporting side effects (Table 5‐D).

## 4 DISCUSSION

This study identified multiple challenges experienced by HCPs involved in HCC care with respect to several critical decision points. In relation to screening and diagnosis, these included: (1) adherence to established screening criteria; (2) use of appropriate imaging for diagnosis; (3) making the decision to perform a diagnostic biopsy. In relation to treatment and management, these critical decision points included: (1) applying guidelines in therapeutic decision‐making; (2) balancing risks/benefits of treatment regimens; (3) managing various patient profiles and comorbidities; (4) optimal sequencing of modalities; and (5) addressing patients' expectations.

The perception that practice guidelines for HCC screening are inadequate has been reported in this study and elsewhere: for example, in a recent evaluation of HCC guidelines by radiation oncologists, the lowest overall score of any of the domains was “applicability,” among the 18 guidelines examined.^26^ This was attributed to the inadequacy of guidelines to adapt to the rapidly‐changing treatment landscape and the necessary multi‐disciplinary nature of HCC care.^26^ A general assessment of international HCC guidelines posits that certain challenges (i.e., regional variations in care practices, resources, disease prevalence) make pursuit of universal, applicable, and reliable HCC guidelines unrealistic.^27^ Existing guidelines are perceived to be inconsistent in relation to recommendations for identifying high‐risk individuals and factoring in the combination of ultrasonography and testing serum AFP levels due to false positives and imprecision.^27^ Some of these inconsistencies relate to lack of high‐level dependable evidence for the efficacy of the proposed interventions. Our study found that there were significant differences among professions and specialties in their perception of guidelines, with HEP and IR finding them less reliable. This may be explained by the different professional roles (i.e., IRs are not expected to be involved directly in the screening of HCC patients) and by recent controversies regarding both the efficacy and best practices for HCC screening that more directly impact these professionals.^28^, ^29^, ^30^ The consequences of inadequate or unclear guidelines also impact primary care providers who play an important role in screening for HCC. A lack of knowledge of HCC screening practices, including misconceptions about HCC surveillance, was reported in a 2019 study of primary care providers.^31^ Despite these challenges, the scope and scale of the issue is becoming better understood. As the quality of evidence needed to support better guidelines improves, knowledge translation and implementation science will help increase awareness and enhance application of the updated guidelines.^27^ Promoting an open exchange of information and experiences between all members of the healthcare team can help deliver a well‐informed, evidence‐based, cohesive care plan for patients with HCC.

In this research, determining an individualized patient care pathway was complicated by challenges specific to interpreting unclear or atypical imaging results. Rao et al. have suggested that confusion over the clinical significance of a lesion or nodule can delay the establishment of a diagnosis and subsequent treatment.^32^ Prolonged delays (>60 days) in establishing an HCC diagnosis in patients with cirrhosis are associated with guideline non‐adherence on the part of the HCP.^33^ We posit that timeliness and access to rigorous multidisciplinary specialist care could mitigate these diagnostic errors and delays and improve patient outcomes. Patients residing in remote or rural areas often have later clinical presentation and decreased survival rates.^34^ However, a case review by an interdisciplinary tumor board has the potential to reduce mortality when completed within 30 days of diagnosis.^35^ In the absence of formal board reviews, creating an environment that promotes and helps to facilitate case discussions between a broad range of members of the healthcare team can be a valuable mechanism to identify diagnostic challenges and pursue appropriate treatment options while minimizing delays.

The increased emphasis on multi‐disciplinary cancer care is likely to reduce overall discrepancies in the level of knowledge of toxicity found between the professions. Patients often present first with hepatic symptoms and are then treated by a hepatology team. Although patients with early‐stage disease being considered for curative intent therapy may not require seeing a medical oncologist, many patients are detected later and should be referred. Many patients present symptoms and imaging data that do not fully reflect HCC diagnostic criteria and thus may not always be referred to an oncologist. This creates siloed care where HCPs are unaware of the need for close collaboration, knowledge of current treatment implications, or the need for effective plans to manage toxicities as a team comprised of oncologists and radiologists, hepatologists, NPs, and PAs.^36^, ^37^ When a multidisciplinary team is in place, it requires coordinated communication and collaboration which can be complex, especially in light of the fact that quite often four or more providers are concurrently involved in decision‐making. In addition to sub‐optimal skills, such collaboration often is complicated by scheduling issues and limited availability.

Interpreting liver imaging results and determining when a biopsy should be done was reported as a challenge. Since biopsy in patients with suspected HCC comes with delays,^38^ studies suggest a multidisciplinary and patient‐specific approach to these decisions based on a combination of screening approaches and methods of collecting patient data.^39^ Clinical decision‐making algorithms could improve screening and identification of HCC, even with atypical presentation, using radiomics and artificial intelligence.^40^ These deep learning technologies aid in decision‐making for suspected HCC lesions that do not fit the Liver Imaging Reporting and Data System [LI‐RADS] criteria.^41^

We found that HCPs have suboptimal skills related to improving patients' QoL and suboptimal confidence in making changes to reduce side effects and promote adherence. A study of patient perspectives indicated they want more information throughout their care and would benefit from the services of patient advocates, especially to assist them in navigating decisions resulting from the often‐difficult side effects of available treatments.^42^ Improvements in HCC management can be addressed through continuing medical education (CME) and continuing professional development (CPD) activities designed to build skill in establishing trust with the patient and setting realistic expectations in terms of potential treatment side effects. Also, the addition of patient advocate resources and improved patient education may assist in building a base of knowledge within the patient and, in so doing, alleviating the impact of HCC on well‐being and autonomy, as well as promoting patient's active participation in the management of the condition.

Recent approvals and improvement in systemic and combination treatment options for HCC^1^, ^43^ occurred during the data collection phase of this study. Regardless, concerns about treatment safety and management of side effects of antineoplastics remain and the addition of new HCC treatment modalities may introduce additional challenges; for example, some patients experience rapid disease progression following treatment with immune checkpoint inhibitors^12^ and decision‐making for each type of treatment becomes more challenging due to a lack of evidence for optimal therapy sequencing.^9^

This study highlighted significant differences among professions in their knowledge of guidelines, plus their level of skill interpreting imaging, treating patients, and ensuring QoL, as some competencies are more‐directly related to some roles as compared to others. This supports the position of previous studies that HCC, due to its nature and complexity, requires a care team with a wide range of knowledge, experience, and competencies within the context of a multidisciplinary approach.^36^, ^37^, ^38^, ^39^, ^40^, ^41^, ^42^, ^43^, ^44^ It is crucial that members of the healthcare team recognize and respect the role that each one plays in delivering optimal patient care.

### 4.1 Limitations

This study's findings stem from self‐reported data rather than empirical observations. To minimize self‐reporting biases such as social desirability,^45^ our methodology included the use of triangulation (i.e., the combination of different data sources, research methodologies and/or interpretation viewpoints in the study of the same phenomenon)^46^ and maximum variation purposive sampling (i.e., where participants are selected to represent a broad spectrum of perspectives).^23^ Results from the qualitative phase may have been limited by the low sample sizes of some sub‐groups (e.g., PAs); however, triangulation with the quantitative findings has ensured the trustworthiness of the overall findings. Caution should be taken when generalizing the findings to other professions/specialties involved in HCC or to other countries. More studies should be done to inform the development of local educational activities/offerings and region‐specific and setting‐specific needs assessments should be conducted to ensure the benefits of developing precise activities for the targeted learners and the needs of the patient population.

## 5 CONCLUSION

This study illuminated the educational needs of providers involved in the spectrum of care of patients with HCC. It highlighted needs to improve the use, content, and knowledge of guidelines for HCC, as well as to enhance skills needed to appropriately screen and diagnose HCC and to improve decision‐making when faced with HCC lesions with atypical presentation. Confidence and skills to enhance QoL were also found to be lacking, despite being critically important for HCC (as a chronic, complex, and severe condition).^47^ Although these challenges could be ameliorated in part by CME/CPD, an emphasis on implementing a multi‐disciplinary team approach to HCC care may be equally integral to improving patient outcomes, as the complicated and diverse presentation of HCC, and the number of HCPs involved in care must be better coordinated to optimize the complementary skills of a diverse team. These findings should be taken into consideration by clinicians in their continuous reflection to improve their practice, and by educators when developing educational interventions on early diagnosis and proper management of patients with HCC, especially as the demand for evidence‐based CME/CPD increases.^48^

## AUTHOR CONTRIBUTIONS

**Ginny Jacobs:** Conceptualization (lead); funding acquisition (equal); investigation (equal); methodology (equal); project administration (equal); supervision (lead); validation (equal); writing – original draft (equal); writing – review and editing (equal). **Deborah A. Boyle:** Methodology (equal); validation (equal); writing – review and editing (equal). **Hashem B. El‐Serag:** Methodology (equal); validation (equal); writing – review and editing (equal). **Robert J Lewandowski:** Methodology (equal); validation (equal); writing – review and editing (equal). **Stacey Stein:** Methodology (equal); validation (equal); writing – review and editing (equal). **Patrice Lazure:** Conceptualization (supporting); data curation (lead); formal analysis (lead); investigation (lead); methodology (equal); project administration (supporting); supervision (equal); validation (equal); visualization (equal); writing – original draft (equal); writing – review and editing (equal). **Pam McFadden:** Conceptualization (equal); funding acquisition (lead); methodology (equal); project administration (equal); supervision (equal); validation (equal); writing – review and editing (equal).

## FUNDING INFORMATION

This study was financially supported by Eisai Co., Ltd.

## CONFLICT OF INTEREST

GJ and PM are employees of AXDEV Global Inc. DAB has nothing to disclose. HBE is supported by NIH P30DK056338. RJL acted as an advisor to BD, Boston Scientific Corporation, Varian, ABK Medical, and Alhambra Medical. SMS has received compensation for advisory roles with Merck, Genentech, Exelixis, QED, and Astra Zeneca. PL is an employee of AXDEV Group Inc. While AXDEV Global and AXDEV Group are private for‐profit companies, they are specialized in educational research with a focus on behavioral and implementation sciences. They do not engage in clinical research or other practice‐related business and, therefore, the employment status of the three AXDEV coauthors does not constitute any conflict of interest with the findings of the study.

## Data Availability

The data that supports the findings of this study are available upon reasonable request to the corresponding author. Due to privacy and ethical restrictions, the data is not publicly available.

## References

[1] Ghavimi S , Apfel T , Azimi H , Persaud A , Pyrsopoulos NT . Management and treatment of hepatocellular carcinoma with immunotherapy: a review of current and future options. J Clin Transl Hepatol. 2020;8:168‐176. 32832397 10.14218/JCTH.2020.00001 PMC7438354

[2] Bray F , Ferlay J , Soerjomataram I , Siegel RL , Torre LA , Jemal A . Global cancer statistics 2018: GLOBOCAN estimates of incidence and mortality worldwide for 36 cancers in 185 countries. Cancer J Clin. 2018;68:394‐424. 10.3322/caac.21492 30207593

[3] Petrick JL , Florio AA , Znaor A , et al. International trends in hepatocellular carcinoma incidence, 1978–2012. Int J Cancer. 2020;147:317‐330. 31597196 10.1002/ijc.32723 PMC7470451

[4] Vo Quang E , Shimakawa Y , Nahon P . Epidemiological projections of viral‐induced hepatocellular carcinoma in the perspective of WHO global hepatitis elimination. Liver Int. 2021;41:915‐927. 33641230 10.1111/liv.14843

[5] Dutta R , Mahato RI . Recent advances in hepatocellular carcinoma therapy. Pharmacol Ther. 2017;173:106‐117. 28174094 10.1016/j.pharmthera.2017.02.010 PMC5777523

[6] Sayiner M , Golabi P , Younossi ZM . Disease burden of hepatocellular carcinoma: a global perspective. Dig Dis Sci. 2019;64:910‐917. 30835028 10.1007/s10620-019-05537-2

[7] Golabi P , Rhea L , Henry L , Younossi ZM . Hepatocellular carcinoma and non‐alcoholic fatty liver disease. Hepatol Int. 2019;13:688‐694. 31701393 10.1007/s12072-019-09995-8

[8] Pazgan‐Simon M , Serafinska S , Janocha‐Litwin J , Simon K , Zuwala‐Jagiello J . Diagnostic challenges in primary hepatocellular carcinoma: case reports and review of the literature. Case Rep Oncol Med 2015;2015: 878763, 1, 5. 25922775 10.1155/2015/878763 PMC4397422

[9] Singal AG , Lampertico P , Nahon P . Epidemiology and surveillance for hepatocellular carcinoma: new trends. J Hepatol. 2020;72:250‐261. 31954490 10.1016/j.jhep.2019.08.025 PMC6986771

[10] Balogh J , Victor D 3rd , Asham EH , et al. Hepatocellular carcinoma: a review. J Hepatocell Carcinoma. 2016;3:41‐53. 27785449 10.2147/JHC.S61146 PMC5063561

[11] Rimassa L , Pressiani T , Merle P . Systemic treatment options in hepatocellular carcinoma. Liver Cancer. 2019;8:427‐446. 31799201 10.1159/000499765 PMC6883446

[12] Forner A , Da Fonseca LG , Díaz‐González Á , Sanduzzi‐Zamparelli M , Reig M , Bruix J . Controversies in the management of hepatocellular carcinoma. JHEP Rep. 2019;1:17‐29. 32039350 10.1016/j.jhepr.2019.02.003 PMC7001551

[13] Russo FP , Imondi A , Lynch EN , Farinati F . When and how should we perform a biopsy for HCC in patients with liver cirrhosis in 2018? A Review. Dig Liver Dis. 2018;50:640‐646. 29636240 10.1016/j.dld.2018.03.014

[14] Kumari R , Sahu MK , Tripathy A , Uthansingh K , Behera M . Hepatocellular carcinoma treatment: hurdles, advances and prospects. Hepat Oncol. 2018;5:HEP08. 31293776 10.2217/hep-2018-0002 PMC6613045

[15] Su T‐S , Liang P , Zhou Y , et al. Stereotactic body radiation therapy vs. transarterial chemoembolization in inoperable Barcelona clinic liver cancer stage a hepatocellular carcinoma: a retrospective, propensity‐matched analysis. *Front* . Oncologia. 2020;10:347. 10.3389/fonc.2020.00347 PMC7105822 32266136

[16] Galle PR , Dufour JF , Peck‐Radosavljevic M , Trojan J , Vogel A . Systemic therapy of advanced hepatocellular carcinoma. Future Oncol. 2021;17:1237‐1251. 33307782 10.2217/fon-2020-0758

[17] Rimola J . Heterogeneity of hepatocellular carcinoma on imaging. Semin Liver Dis. 2020;40:61‐69. 31266063 10.1055/s-0039-1693512

[18] Yang JD , Heimbach JK . New advances in the diagnosis and management of hepatocellular carcinoma. BMJ. 2020;371:m3544. 33106289 10.1136/bmj.m3544

[19] Laube R , Sabih A‐H , Strasser SI , Lim L , Cigolini M , Liu K . Palliative care in hepatocellular carcinoma. J Gastroenterol Hepatol. 2021;36:618‐628. 32627853 10.1111/jgh.15169

[20] Turner SF , Cardinal LB , Burton RM . Research design for mixed methods: a triangulation‐based framework and roadmap. Organ Res Methods. 2017;20:243‐267.

[21] ICC/ESOMAR . ICC/ESOMAR (International Chamber of Commerce/European Society for Opinion and Marketing Research) International code on market, opinion and social research and data analytics 2016. Available from URL: https://www.esomar.org/what‐we‐do/code‐guidelines.

[22] Persad G , Fernandez Lynch H , Largent E . Differential payment to research participants in the same study: an ethical analysis. J Med Ethics. 2019;45:318‐322. 30846490 10.1136/medethics-2018-105140

[23] Palinkas LA , Horwitz SM , Green CA , Wisdom JP , Duan N , Hoagwood K . Purposeful sampling for qualitative data collection and analysis in mixed method implementation research. Adm Policy Ment Health. 2015;42:533‐544. 24193818 10.1007/s10488-013-0528-y PMC4012002

[24] Assarroudi A , Heshmati Nabavi F , Armat MR , Ebadi A , Vaismoradi M . Directed qualitative content analysis: the description and elaboration of its underpinning methods and data analysis process. J Res Nurs. 2018;23:42‐55. 34394406 10.1177/1744987117741667 PMC7932246

[25] Dixon‐Woods M , Agarwal S , Jones D , Young B , Sutton A . Synthesising qualitative and quantitative evidence: a review of possible methods. J Health Serv Res Policy. 2005;10:45‐53. 15667704 10.1177/135581960501000110

[26] Rim CH , Cheng J , Huang W‐Y , et al. An evaluation of hepatocellular carcinoma practice guidelines from a radiation oncology perspective. Radiother Oncol. 2020;148:73‐81. 32335365 10.1016/j.radonc.2020.03.027

[27] Yu SJ . A concise review of updated guidelines regarding the management of hepatocellular carcinoma around the world: 2010‐2016. Clin Mol Hepatol. 2016;22:7‐17. 27044761 10.3350/cmh.2016.22.1.7 PMC4825164

[28] Tayob N , Corley DA , Christie I , et al. Validation of the updated hepatocellular carcinoma early detection screening algorithm in a community‐based cohort of patients with cirrhosis of multiple etiologies. Clin Gastroenterol Hepatol. 2021;19(1443–1450):e1446. 10.1016/j.cgh.2020.07.065 PMC8201947 32768590

[29] Zhang J , Chen G , Zhang P , et al. The threshold of alpha‐fetoprotein (AFP) for the diagnosis of hepatocellular carcinoma: a systematic review and meta‐analysis. PLoS One. 2020;15:e0228857. 32053643 10.1371/journal.pone.0228857 PMC7018038

[30] Koulouris A , Tsagkaris C , Spyrou V , Pappa E , Troullinou A , Nikolaou M . Hepatocellular carcinoma: an overview of the changing landscape of treatment options. J Hepatocell Carcinoma. 2021;8:387‐401. 34012929 10.2147/JHC.S300182 PMC8128500

[31] Simmons OL , Feng Y , Parikh ND , Singal AG . Primary care provider practice patterns and barriers to hepatocellular carcinoma surveillance. Clin Gastroenterol Hepatol. 2019;17:766‐773. 30056183 10.1016/j.cgh.2018.07.029 PMC7212522

[32] Rao A , Rich NE , Marrero JA , Yopp AC , Singal AG . Diagnostic and therapeutic delays in patients with hepatocellular carcinoma. J Natl Compr Canc Netw. 2021;1:1‐14. 10.6004/jnccn.2020.7689 34077908

[33] Choi DT , Davila JA , Sansgiry S , et al. Factors associated with delay of diagnosis of hepatocellular carcinoma in patients with cirrhosis. Clin Gastroenterol Hepatol. 2020; 19:1679‐1687. 32693047 10.1016/j.cgh.2020.07.026 PMC7855025

[34] Taye BW , Clark PJ , Hartel G , Powell EE , Valery PC . Remoteness of residence predicts tumor stage, receipt of treatment, and mortality in patients with hepatocellular carcinoma. JGH Open. 2021;5:754‐762. 34263069 10.1002/jgh3.12580 PMC8264246

[35] Serper M , Taddei TH , Mehta R , et al. Association of provider specialty and multidisciplinary care with hepatocellular carcinoma treatment and mortality. Gastroenterology. 2017;152:1954‐1964. 28283421 10.1053/j.gastro.2017.02.040 PMC5664153

[36] Meriggi F , Graffeo M . Clinical characterisation and management of the main treatment‐induced toxicities in patients with hepatocellular carcinoma and cirrhosis. Cancer. 2021;13:584. 10.3390/cancers13030584 PMC7867371 33540870

[37] Cui T‐M , Liu Y , Wang J‐B , Liu L‐X . Adverse effects of immune‐checkpoint inhibitors in hepatocellular carcinoma. OncoTargets Ther. 2020;13:11725‐11740. 10.2147/OTT.S279858 PMC7678689 33235462

[38] Rho YS , Pagano I , Wong LL , Kwee SA , Acoba JD . Factors and survival implications associated with biopsy of hepatocellular carcinoma. HPB (Oxford). 2020; 23:1054‐1060. 33229278 10.1016/j.hpb.2020.11.001

[39] Kim JH , Joo I , Lee JM . Atypical appearance of hepatocellular carcinoma and its mimickers: how to solve challenging cases using gadoxetic acid‐enhanced liver magnetic resonance imaging. Korean J Radiol. 2019;20:1019‐1041. 31270973 10.3348/kjr.2018.0636 PMC6609440

[40] Mokrane F‐Z , Lu L , Vavasseur A , et al. Radiomics machine‐learning signature for diagnosis of hepatocellular carcinoma in cirrhotic patients with indeterminate liver nodules. Eur Radiol. 2020;30:558‐570. 31444598 10.1007/s00330-019-06347-w

[41] Oestmann PM , Wang CJ , Savic LJ , et al. Deep learning–assisted differentiation of pathologically proven atypical and typical hepatocellular carcinoma (HCC) versus non‐HCC on contrast‐enhanced MRI of the liver. Eur Radiol. 2021;31:4981‐4990. 33409782 10.1007/s00330-020-07559-1 PMC8222094

[42] Gill J , Baiceanu A , Clark PJ , et al. Insights into the hepatocellular carcinoma patient journey: results of the first global quality of life survey. Future Oncol. 2018;14:1701‐1710. 29543521 10.2217/fon-2017-0715

[43] FDA approves atezolizumab plus bevacizumab for unresectable hepatocellular carcinoma. Available from URL: https://www.fda.gov/drugs/resources‐information‐approved‐drugs/fda‐approves‐atezolizumab‐plus‐bevacizumab‐unresectable‐hepatocellular‐carcinoma.

[44] Byrd K , Alqahtani S , Yopp AC , Singal AG . Role of multidisciplinary care in the management of hepatocellular carcinoma. Semin Liver Dis. 2021;41(1):1‐8. 33764480 10.1055/s-0040-1719178

[45] Althubaiti A . Information bias in health research: definition, pitfalls, and adjustment methods. J Multidiscip Healthc. 2016;9:211‐217. 27217764 10.2147/JMDH.S104807 PMC4862344

[46] Smith J , Noble H . Bias in research. Evid Based Nurs. 2014;17:100‐101. 25097234 10.1136/eb-2014-101946

[47] Colagrande S , Inghilesi AL , Aburas S , Taliani GG , Nardi C , Marra F . Challenges of advanced hepatocellular carcinoma. World J Gastroenterol. 2016;22:7645‐7659. 27678348 10.3748/wjg.v22.i34.7645 PMC5016365

[48] Filipe HP , Mack HG , Golnik KC . Continuing professional development: progress beyond continuing medical education. Ann Eye Sci. 2017;2:46.

